# MicroRNA‐30e‐5p promotes cell growth by targeting *PTPN13* and indicates poor survival and recurrence in lung adenocarcinoma

**DOI:** 10.1111/jcmm.13198

**Published:** 2017-06-27

**Authors:** Li Zhuang, Tao Shou, Ke Li, Chun‐Lin Gao, Lin‐Can Duan, Li‐Zhou Fang, Qin‐Yong Zhang, Zong‐Ning Chen, Chao Zhang, Shou‐Tao Yang, Gao‐Feng Li

**Affiliations:** ^1^ Department of Palliative Medicine Palliative Medicine Research Center The Third Affiliated Hospital of Kunming Medical University Kunming Yunnan Province China; ^2^ Department of Oncology The First People's Hospital of Yunnan Province Kunming Yunnan Province China; ^3^ The Second Department of Medicine The Third Affiliated Hospital of Kunming Medical University Kunming Yunnan Province China; ^4^ Department of Thoracic Surgery The Third Affiliated Hospital of Kunming Medical University Kunming Yunnan Province China; ^5^ The Second Department of Respiratory The First Affiliated Hospital of Kunming Medical University Kunming Yunnan Province China; ^6^ Department of Pharmacy The Third Affiliated Hospital of Kunming Medical University Kunming Yunnan Province China; ^7^ Department of Cardiology The People's Hospital of Lijiang City Lijiang Yunnan Province China; ^8^ Cancer Treatment Center The First Affiliated Hospital of Kunming Medical University Kunming Yunnan Province China; ^9^ Department of Nursing The Third Affiliated Hospital of Kunming Medical University Kunming Yunnan Province China

**Keywords:** microRNA‐30e‐5p, lung adenocarcinoma, growth, *PTPN13*

## Abstract

Aberrant microRNA expression is involved in the regulation of various cellular processes, such as proliferation and metastasis in multiple diseases including cancers. MicroRNA‐30e‐5p (miR‐30e) was previously reported as an oncogenic or tumour suppressing miRNA in some malignancies, but its function in lung adenocarcinoma (LAC) remains largely undefined. In this study, we found that the expression of miR‐30e was increased in LAC tissues and cell lines, associated with tumour size and represented an independent prognostic factor for overall survival and recurrence of LAC patients. Further functional experiments showed that knockdown of miR‐30e suppressed cell growth while its overexpression promoted growth of LAC cells and xenografts *in vitro* and *in vivo*. Mechanistically, *PTPN13* was identified as the direct target of miR‐30e in LAC, in which *PTPN13* expression was down‐regulated in LAC tissues and showed the inverse correlation with miR‐30e expression. Overexpression of *PTPN13* inhibited cell growth and rescued the proliferation‐promoting effect of miR‐30e through inhibition of the *EGFR* signalling. Altogether, our findings suggest that miR‐30e could function as an oncogene in LAC *via* targeting *PTPN13* and act as a potential therapeutic target for treating LAC.

## Introduction

Lung cancer is presently the leading cause of cancer deaths worldwide with the highest mortality rate 1. Non‐small cell lung cancer (NSCLC) including the LAC accounts for approximately 80% of lung cancer cases 2. Despite recent advances in the treatment of LAC including surgical resection, chemotherapy, radiation therapy or a combination of targeted therapy, the survival and prognosis of advanced NSCLC patients remain unsatisfactory with 5‐year survival rate less than 20% 3. Therefore, identification of new prognostic biomarkers related to LAC tumorigenesis and progression is urgently needed.

MicroRNAs as small non‐coding RNAs of approximately 18–25 nucleotides play critical roles in post‐transcriptional gene regulation in various diseases. One particular microRNA, miR‐30e, was previously reported to be implicated in regulating vascular diseases such as neointimal hyperplasia by targeting Ca(2+)/calmodulin‐dependent protein kinase IIδ 4 and mesenchymal stem cells and aortic smooth muscle cells by targeting *IGF2* 5. Based on different tumour histopathological types, miR‐30e showed differential expression in different cancer types. It was found down‐regulated in bladder cancer 6, breast cancer 7, 8 and rectal adenocarcinoma without chemoradiotherapy responsiveness 9 and correlated with the clinical stage and favourable prognosis, indicating the tumour suppressive role in cancer. But, some studies showed that miR‐30e was up‐regulated in malignant salivary gland tumours 10, LAC 11 involving cancer invasion and metastasis 12, revealing miR‐30e as the potential diagnostic and prognostic biomarker for the therapy of these malignancies.

In spite of a small amount of data displaying the decreased expression of miR‐30e in NSCLC 13, 14, increasing evidence showed miR‐30e was up‐regulated in cancers including LAC 11 and exerted the oncogenic role in cancer 10, 12. The functions and molecular regulatory mechanisms of miR‐30e in LAC remain unknown, in this study, we found that miR‐30e expression was increased in LAC tissues and cell lines and indicated poor survival and recurrence in LAC patients. In addition, miR‐30e overexpression promoted growth of LAC cells *in vitro* and *in vivo* by targeting *PTPN13*.

## Materials and methods

### Materials

Human LAC tissues were collected from The Third Affiliated Hospital of Kunming Medical University. LAC cell lines (BEAS‐2B, A549. NCI‐2228, NCI‐H23, NCI‐H2085, NCI‐H2087. NCI‐H1993, NCI‐H522 and NCI‐H1975) used in our experiments were from Chinese Academy of Science Shanghai Cell Bank. Lentivirus‐mediated miR‐30e overexpression, miR‐30e shRNA and *PTPN13* overexpression vectors, negative control vector (NC) and virion‐packaging elements were from Genechem (Shanghai, China); The primary antibodies of PTPN13 (rabbit monoclonal antibody, ab198882), β‐actin (mouse monoclonal antibody, ab8226), EGFR (rabbit monoclonal antibody, ab52894), AKT(rabbit polyclonal antibody, ab126811), p‐AKT(rabbit polyclonal antibody, ab18206) were from Abcam (Cambridge, MA, USA). The horseradish peroxidase‐linked second goat antibody was from Sigma Corporation (St Louis, MO, USA). Dulbecco's modified Eagle medium (DMEM) and foetal bovine serum (FBS) were from Thermo Fisher Scientific Inc (Waltham, MA, USA); 3‐(4,5)‐dimethylthiahiazo (‐z‐yl)‐3,5‐ di‐phenytetrazoliumromide (MTT) was from Dingguo biology (Shanghai, China); TRIzol reagent and lipofectamine 2000 were from Invitrogen (Carlsbad, CA, USA); M‐MLV Reverse Transcriptase was from Promega (Madison, WI, USA); SYBR Green Master Mixture was from Takara (Otsu, Japan). ECL‐PLUS/Kit was from GE Healthcare (Piscataway, NJ, USA).

### Clinical samples

LAC and corresponding adjacent normal tissues were collected from patients undergoing resection of primary LAC in a total of 78 consecutive cases admitted in our hospital from January 2007 to December 2015. Overall survival (OS) was defined as the interval between the dates of surgery and death. The LAC subtypes classification was implemented according to the guidance of 2015 WHO new classification criteria of lung tumours 15. Written consents approving the use of LAC tissues for research purposes were acquired from the patients or their parents before sample collection. The study protocol was approved by Medical Ethics Committee of The Third Affiliated Hospital of Kunming Medical University. The medical records of the patients were listed in Table S1.

### Construction of vectors

A fragment of miR‐30e was generated using the following primers: sense, 5′‐TGTAAACATCCTTGACTGGAAG‐3′ and antisense, 5′‐GCGAGCACAG AATTAATACGAC‐3′ and inserted into the pMD‐18T vector with a green fluorescent protein reporter gene within the EcoRI/XhoI restriction sites. The aforementioned miR‐30e plasmid pCDNA3‐GFP was transfected into 293T cells, and the lentiviral particle‐enriched supernatant was obtained 48 hrs later. A scrambled sequence was used as a scrambled negative control.

### Cell culture and transfection

LAC cells were cultured in DMEM medium supplemented with 10% heat‐inactivated FBS, 100U/ml of penicillin and 100 μg/ml of streptomycin. Cells in this medium were placed in a humidified atmosphere containing 5% CO_2_ at 37°C. LAC cells were transfected with experimental virus or control virus and cultured at 37°C and 5% CO_2_ for 6 hrs. Then supernatant was discarded, and serum containing growth medium was added. Positive and stable transfectants were selected and expanded for further study.

### Quantitative Real‐time PCR

To quantitatively confirm the expression levels of miR‐30e in LAC tissues, real‐time PCR was performed. Total RNA was extracted from each clone using TRIzol according to the manufacturer's protocol. Reverse transcription was carried out using M‐MLV, and cDNA amplification was performed using the SYBR Green Master Mix kit (Takara, Otsu, Japan) according to the manufacturer's guidelines. The primer sequences for miR‐30e and *PTPN13* were listed as follows: miR‐30e Forward: 5′ ‐GGCGTGTAAACATCCTT GACTG‐3′, Reverse: 5′‐GTGCAGGGTCCGAGGT‐3′ (62 bp); U6 Forward:5′‐GCTT CGGCAGCACATATACTAAAAT‐3′, Reverse: 5′‐CGCTTCACGAATTTGCGTG TCAT‐3′. *PTPN13* forward 5‐TGGCTCTC CAGGCTGAGTATG‐3′ and reverse 5′‐CGGGCAAATAGTGCTCCATT‐3′; *GAPDH*, Forward: 5′‐AACTTTGGGATTGTGGAAGG‐3′, Reverse: 5′‐ACACA TTGGGGGTAGGAACA ‐3′. *GAPDH* gene and U6 was used as an endogenous control. Data were analysed using the comparative Ct method. Three separate experiments were performed for each clone.

### Western blot analysis

LAC cells were harvested and extracted using lysis buffer (100 mM Tris–HCl, 2% SDS, 1 mM mercaptoethanol, 25% glycerol). Cell extracts were boiled in loading buffer, and then, equal amount of cell extracts was separated on 15% SDS‐PAGE gels. Separated protein bands were transferred into polyvinylidene fluoride (PVDF) membranes. The primary antibodies were diluted at a ratio of 1:1000 according to the instructions and incubated overnight at 4°C. Horseradish peroxidase‐linked secondary antibodies were added at a dilution ratio of 1:5000 and incubated at room temperature for 2 hrs. The membranes were washed with PBS for three times, and the immunoreactive bands were visualized using ECL‐PLUS/Kit (GE Healthcare) according to the kit's instruction. Three separate experiments were performed for each clone.

### Cell viability assay

Cell proliferation was analysed by the MTT assay. LAC cells transfected with miR‐30e or *PTPN13* were incubated in 96‐well plates at a density of 4 × 10^3^ cells per well with DMEM medium supplemented with 10% FBS. Cells were treated with 20 μl of MTT and subsequently incubated with 150 μl of DMSO for 10 min. The colour reaction was measured at 570 nm using an Enzyme Immunoassay Analyzer (Bio‐Rad, Hercules, CA, USA). Each experiment was repeated for three times.

### Colony formation assay

2× DMEM containing 20% FBS and 2 × 10^3^ cells was mixed with equal volume of 0.7% agarose and immediately plated in 6‐well plates containing an underlayer of 0.5% agarose made in 1× DMEM supplemented with 10% FBS. The plates were cultured at 37°C under 5% CO_2_ for 7 days. Each experiment was repeated for three times.

### The target screening of miR‐30e in cancer tissues

To clarify the molecular mechanisms by which miR‐30e regulates cell growth in LAC cells, we identified the potential target genes of miR‐30e using the starBase v2.0 in the website (http://starbase.sysu.edu.cn) and the strict screening conditions including three target prediction algorithms (targetScan, PITA and miRanda), very high stringency (>5) and being expressed in at least three or more tumours in starBase v2.0 were required to predict the target genes of miR‐30e in cancer tissues.

### Dual‐luciferase reporter assay

LAC cells were seeded into 24‐well plates. After 24‐hr incubation, 1 mg pmirGLO report vector carrying wild‐type 3′‐UTR or mutated 3′‐UTR of miR‐30e target was co‐transfected with 100 pmol miR‐30e or NC(Negative Control) into the LAC cells. 48 hrs after transfection, fiefl and renilla luciferase activities were examined with a Dual‐luciferase Reporter System (Promega). PmirGLO report vector was used as a positive control.

### Animal experiments

Six‐week‐old female immune‐deficient nude mice (BALB/c‐nu) were bred at the laboratory animal facility. All experimental procedures were performed according to the regulations and internal biosafety and bioethics guidelines of The Third Affiliated Hospital of Kunming Medical University and the Kunming Municipal Science and Technology Commission. Six mice were randomly divided into two groups. The three mice in experimental group were injected subcutaneously with 1 × 10^6^ LAC A549 cell line stably transfected with miR‐30e. The other three mice in control group were injected subcutaneously with 1 × 10^6^ LAC A549 cell line stably transfected with miR‐NC. Mice were monitored daily and developed a subcutaneous tumour. The tumour volume every 3 days was measured with a calliper using the formula:

$$\text{volume}={\left(\text{length}\times \text{width}\right)}^{2}/2$$

### Statistical analysis

SPSS 20.0 (St Louis, MO, USA) was used for the statistical analysis. All of the values were recorded as the Mean ± S.E.M. from at least three independent experiments. Two‐tailed Student's *t*‐test was used to evaluate the differences between each group. Survival curves were plotted using the Kaplan–Meier method and assessed for the statistical significance using a log‐rank test. Statistical significance was set at *P* < 0.05.

## Result

### Upregulation of miR‐30e in human LAC tissues

**Figure 1:**
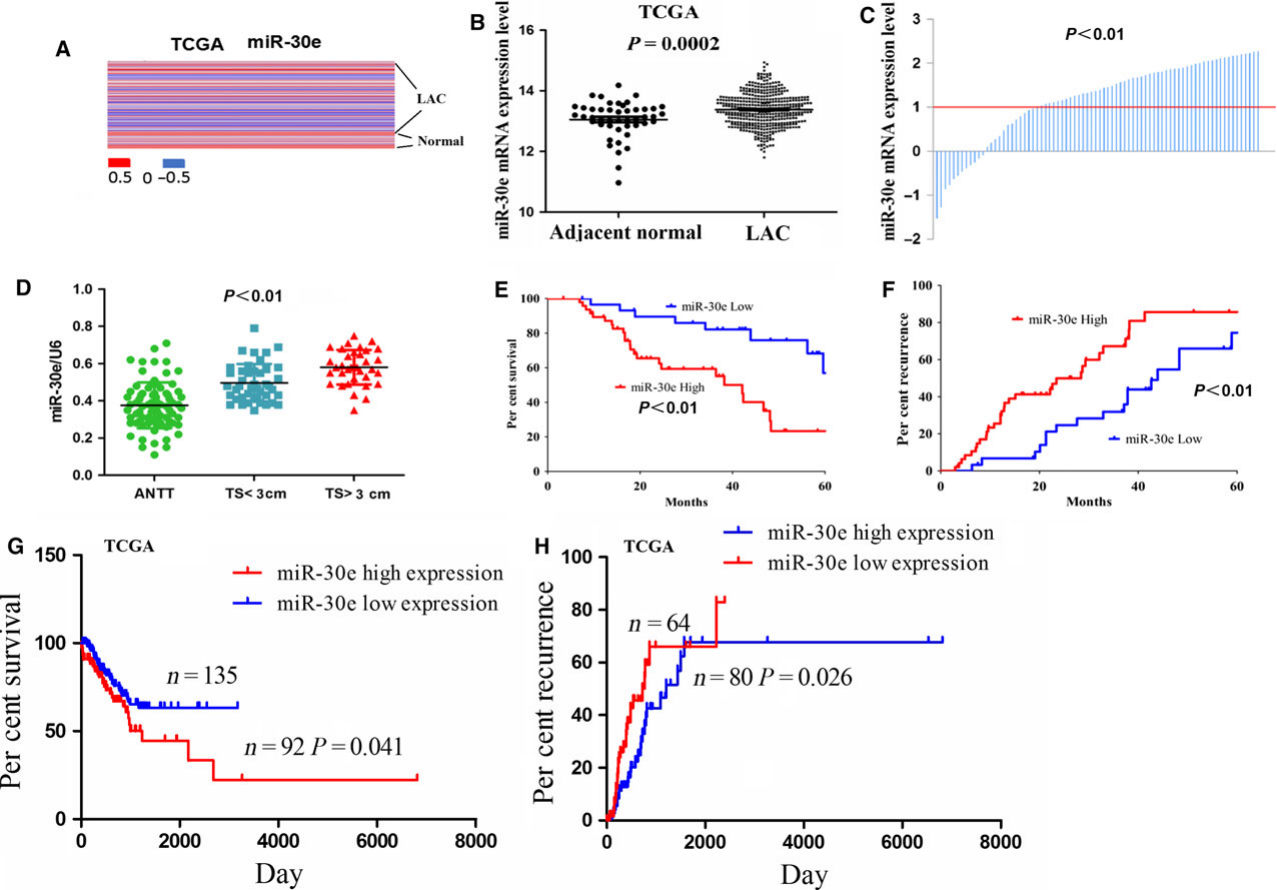
The expression of miR‐30e was up‐regulated in LAC. (**A**) miR‐30e had the differential expression in 452 LAC patient samples and 46 adjacent normal tissues indicated by a 2015 TCGA data set. (**B**) TCGA showed that miR‐30e expression was increased in LAC compared to the adjacent normal tissues. The ordinate value reflected the relative ratio of miR‐30e expression in LAC tissue *versus* adjacent normal tissue. (**C**) miR‐30e expression was significantly up‐regulated in 67.95% (53/78) of LAC patient tissues compared to the adjacent normal. (**D**) The expression level of miR‐30e in LAC patients with tumour size≥3 cm and tumour size<3 cm compared with the adjacent non‐tumour tissues (ANTT). (**E, G**) Overall survival and (**F, H**) recurrence curves demonstrated the correlation of miR‐30e high expression or low expression with the percent survival and recurrence in LAC patients.

Different expression levels of miR‐30e have been reported in lung cancer 12, 14, 16. To evaluate the expression level of miR‐30e in LAC tissues, we performed *in silico* analyses of the differential expression of miR‐30e between 452 LAC patient samples and 46 adjacent non‐cancerous tissues (ANCT) using a 2015 The Cancer Genome Atlas (TCGA) data set (Fig. 1A), showing that miR‐30e expression was increased in LAC samples compared with the adjacent normal (*P* = 0.0002, Fig 1B). We further accomplished the quantitative real‐time PCR in 78 cases of LAC tissues and corresponding adjacent normal tissues. According to the ratio of 2^−▵CT^_LAC_ and 2^−▵CT^_Normal_ in each case, if the value ≧ 1, the expression level of miR‐30e was defined as the high expression; otherwise, it was considered as low expression. Based on this, we found that miR‐30e expression was significantly elevated in 67.95% (53/78) of LAC tissues (*P* < 0.01; Fig. 1C), and the expression level of miR‐30e was up‐regulated in patients with tumour size ≥3 cm compared with those with tumour size <3 cm (*P* < 0.01; Fig. 1D).

### Association of miR‐30e expression with clinicopathological characteristics and prognosis in LAC patients

To demonstrate the correlation of miR‐30e expression with LAC patients, we investigated the correlations between miR‐30e expression and clinicopathological characteristics and prognosis in LAC patients. As indicated in Table 1, the results showed that miR‐30e expression was significantly correlated with tumour size (*P* = 0.028), but had no correlation with age, sex, smoking status, adenocarcinoma subtype, pathological stage and pathological TNM stage in LAC patients (each *P* > 0.05). Meanwhile, TCGA data indicated that miR‐30e high expression had no link with age, gender, smoking status, tumour size, adenocarcinoma subtype, pathological stage and pTNM stage (each *P* > 0.05, Table S2). We further investigated the correlation between miR‐30e expression and overall survival (OS) and recurrence in LAC patients using Kaplan–Meier and multivariate analysis, which indicated that miR‐30e expression was the independent prognostic factor for the OS (*P* = 0.024, Table S3) and recurrence (*P* = 0.036, Table S4) in LAC patients. Overall survival and recurrence curves in our cohort and TCGA cohort demonstrated that LAC patients with high miR‐30e expression displayed lower survival rate (*P* < 0.05, Fig. 1E and G) and higher recurrence rate (*P* < 0.05, Fig. 1F and H) compared to those with miR‐30e low expression. Our results suggested that miR‐30e might be an independent prognostic factor for OS and recurrence of LAC patients in our cohort rather than TCGA cohort.

**Table 1:** The correlation of miR‐30e expression with clinicopathologic characteristics of LAC patients

Variables	Cases (*n*)	miR‐30e		*P* value
		High	Low	
Total	78	53	25	
Age (years)				
≥60	32	24	8	0.269
<60	46	29	17	
Sex				
Male	51	35	16	0.861
Female	27	18	9	
Smoking status				
Current smokers	12	7	5	0.697
Previous smokers	45	32	13	
Slight/No smokers	21	14	7	
Tumour size (cm)				
≥3	36	29	7	0.028
<3	42	24	18	
Adenocarcinoma subtypes				
Adenocarcinoma *in situ* (AIS)	3	0	3	
Minimally invasive adenocarcinoma (MIA)	7	3	4	
Invasive adenocarcinoma				
Lepidic predominant	37	28	9	0.067
Acinar predominant	14	9	5	
Papillary predominant	11	9	2	
Variants of invasive adenocarcinoma				
Invasive mucinous adenocarcinoma (IMA)	6	4	2	
Pathological stage				
I/II	57	36	21	0.138
Ш/IV	21	17	4	
pT stage				
T1/T2	66	46	20	0.441
T3/T4	12	7	5	
pN stage				
Negative	43	29	14	0.916
Positive	35	24	11	
pM stage				
Negative	69	48	21	0.400
Positive	9	5	4	

### Inhibition of cell proliferation and colony formation by miR‐30e knockdown

**Figure 2:**
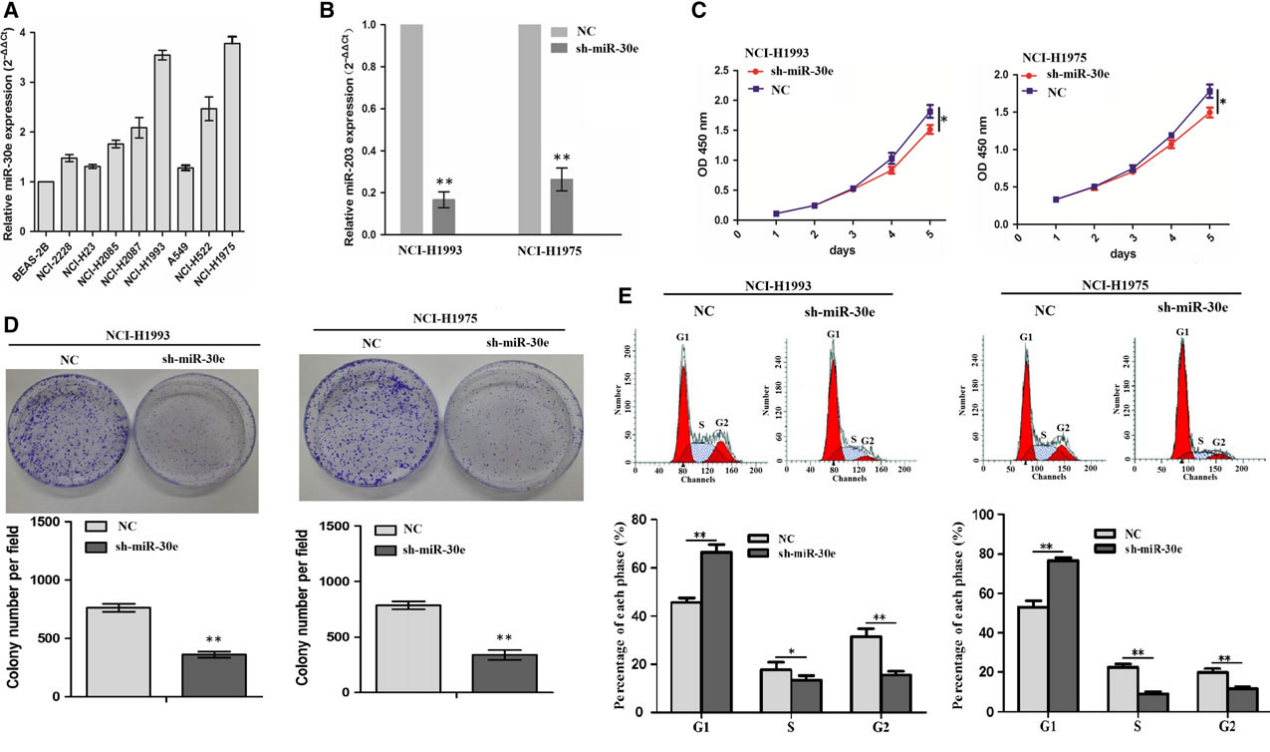
Knockdown of miR‐30e inhibited cell proliferation and colony formation. (**A**) The expression levels of miR‐30e in different LAC cell lines. (**B**) The expression level of miR‐30e was measured by real‐time PCR. (**C**) The effects of miR‐30e knockdown on cell proliferation by MTT assay. (**D**) The effects of miR‐30e knockdown on cell colony formation. (**E**) The effects of miR‐30e knockdown on cell cycle distribution. *P < 0.05, **P < 0.01.

Having confirmed the positive correlation of miR‐30e expression with tumour size in LAC patients (Table 1), we then explored the functions of miR‐30e in LAC. We first examined the differential expression levels of miR‐30e in different LAC cell lines. miR‐30e was found up‐regulated in lung cancer cell lines compared to the BEAS‐2B lung normal cell line, and relatively it had the low expression in A549 and NCI‐H23 cell lines but had the high expression in NCI‐H1993 and NCI‐H1975 cell lines (Fig. 2A). Then, lentivirus‐mediated miR‐30e shRNA was used to transfect into miR‐30e highly expressed NCI‐H1993 and NCI‐H1975 cell lines. After transfection for 48 hrs, miR‐30e expression level was increased in miR‐30e group compared to NC group (**P < 0.01; Fig. 2B). MTT and colony formation assays demonstrated that knockdown of miR‐203 significantly suppressed cell proliferation (Fig. 2C) and colony formation (Fig. 2D) in NCI‐H1993 and NCI‐H1975 cells (**P < 0.01). Flow‐cytometric analysis showed that the proportion of cells at G1 phase increased while that at S and G2 phase decreased after knockdown of miR‐203 in NCI‐H1993 and NCI‐H1975 cells (*P < 0.05, **P < 0.01; Fig. 2E).

Furthermore, miR‐30e overexpression vector was used to transfect into A549 and NCI‐H23 cell lines. After transfection for 48 hrs, miR‐30e expression level was found increased in miR‐30e group compared to NC group (**P < 0.01; Fig. S1A). MTT assay showed that enforced expression of miR‐30e promoted cell proliferation (Fig. S1B and C) in A549 and NCI‐H23 cell lines.

### *PTPN13* as a direct target of miR‐30e in LAC

**Figure 3:**
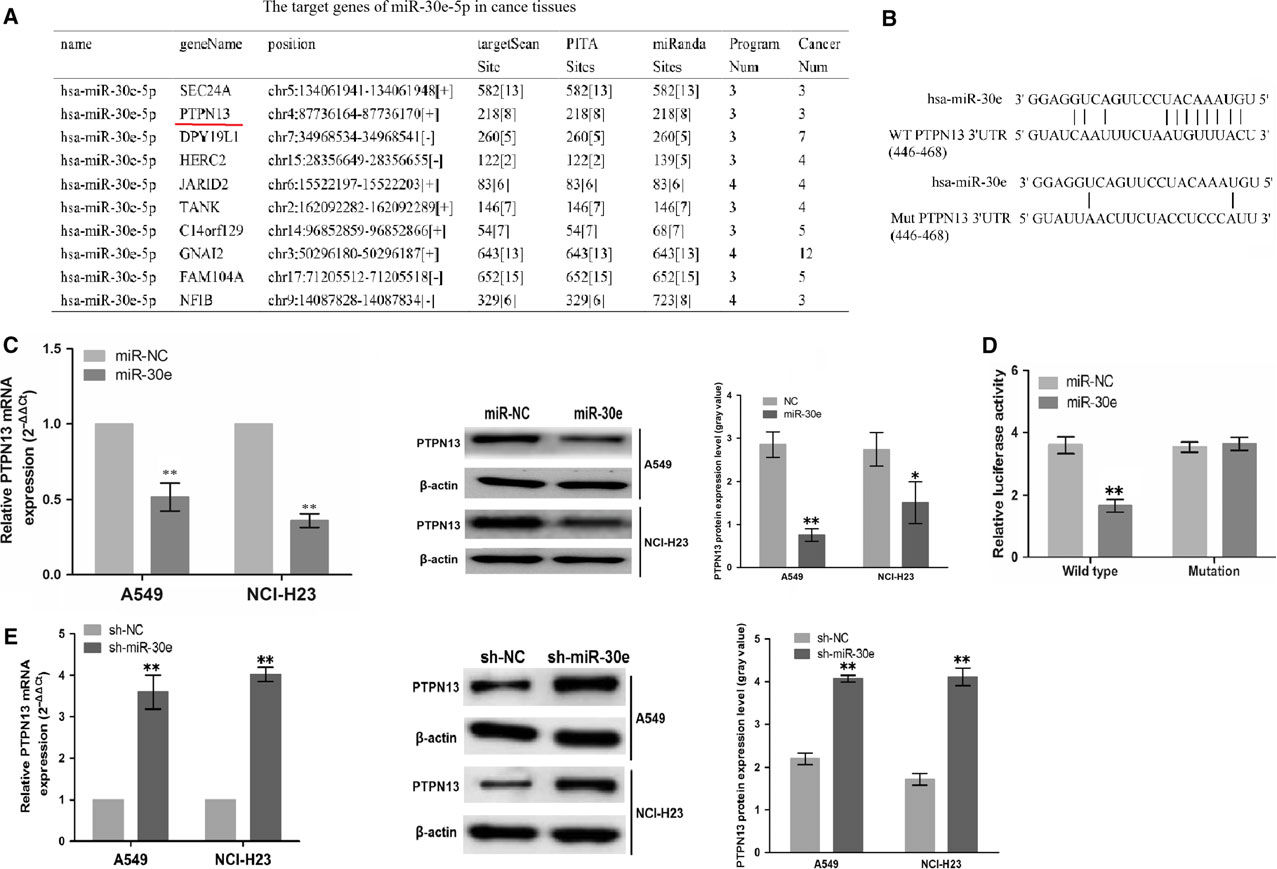
*PTPN13* was identified as a direct target of miR‐30e. **(A)** The main target genes of miR‐30e in cancer tissues**. (B)** Diagrams showed the miR‐30e putative binding sites and corresponding mutant sites of *PTPN13*. (**C**) The expression level of *PTPN13* was examined after transfection with miR‐30e by real‐time PCR and Western blotting assays. (**D**) Luciferase activity was detected after miR‐30e transfection. **(E)** The expression level of *PTPN13* was examined after transfection with sh‐miR‐30e by real‐time PCR and Western blotting assays. *P < 0.05, **P < 0.01.

To clarify the molecular mechanisms by which miR‐30e regulates cell growth in LAC cells, we identified the potential target genes of miR‐30e using the starBase v2.0 in the website (http://starbase.sysu.edu.cn) and the strict screening conditions including three target prediction algorithms (targetScan, PITA and miRanda), very high stringency (>5) and being expressed in at least three or more tumour types in starBase v2.0 were required to predict the target genes of miR‐30e in cancer tissues. As shown in Figure 3A and Table S5, among 164 selected target genes, *PTPN13* was identified as the most suitable candidate target gene duo to its high binding capacity with miR‐30e. To further affirm whether miR‐30e directly binds with the 3′ UTR of *PTPN13*, the WT 3′ UTR or the mutant 3′ UTR target sequences of *PTPN13* (Fig. 3B) were cloned into the luciferase reporter vectors, which were transfected into A549 and NCI‐H23 cells. Our results indicated that miR‐30e decreased the mRNA and protein expression level of *PTPN13* (**P < 0.01, Fig. 3C) and inhibited the luciferase activity of WT 3` UTR of *PTPN13* compared to the Mutation 3` UTR of *PTPN13* in LAC cells (**P < 0.01, Fig. 3D). We also found that knockdown of miR‐30e increased the mRNA and protein expression level of *PTPN13* (**P < 0.01, Fig. 3E).

### Inhibition of cell proliferation and colony formation by *PTPN13*

**Figure 4:**
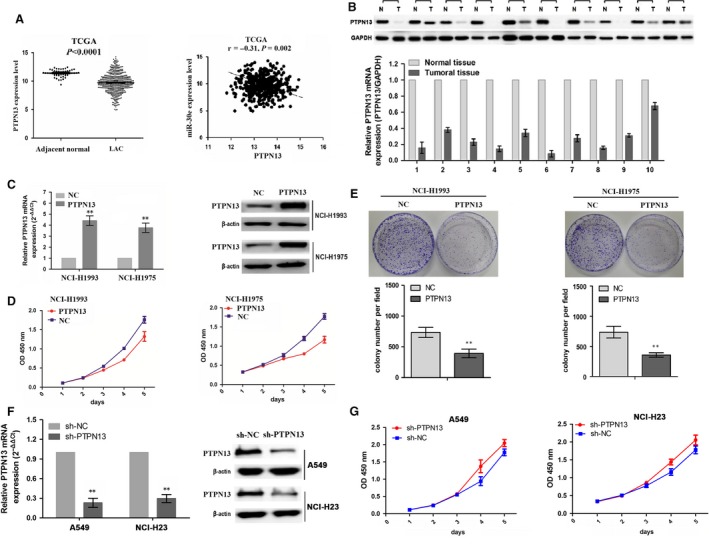
Enforced expression of *PTPN13* inhibited cell proliferation and colony formation. (**A**) TCGA showed a decreased expression of *PTPN13* in LAC compared to the adjacent normal tissues, and a negative correlation of *PTPN13* with miR‐30E expression in LAC. The expression levels of *PTPN13* were determined in human LAC tissues (**B**) and cells **(C)** by real‐time PCR and Western blot assays. (**D**) The effects of *PTPN13* overexpression on cell proliferation by MTT assay. **(E)** The effects of *PTPN13* overexpression on cell colony formation. (**F**) The effect of sh‐PTPN13 vector transfection for 48 hrs on PTPN13 mRNA and protein expression. **(G)** The effects of *PTPN13* depletion on cell proliferation by MTT assay. **P < 0.01.

To realize the expression of *PTPN13* in LAC, we made *in silico* analyses of the differential expression of *PTPN13* between 513 cases of LAC samples and 58 adjacent normal samples by TCGA data set, indicating that *PTPN13* expression was markedly decreased in LAC compared to adjacent normal and had the inverse correlation with the miR‐30e expression (*n* = 452) (Fig. 4A). We found that the mRNA and protein levels of PTPN13 in LAC tissue were lower than those in adjacent normal tissue in 10 representative patients (Fig. 4B). To expound the functions of *PTPN13* in LAC, MTT and colony formation assays were performed. We first proved that *PTPN13* expression was effectively elevated in mRNA and protein levels after *PTPN13* vector transfection for 48 hrs (Fig. 4C). Then, enforced expression of *PTPN13* remarkably decreased cell proliferation (Fig. 4D) and colony formation in LAC cells (Fig. 4E). We also proved that *PTPN13* expression decreased in mRNA and protein levels after sh‐*PTPN13* vector transfection for 48 hrs (Fig. 4F). Then, knockdown of *PTPN13* remarkably promoted cell proliferation (Fig. 4G).

### Tumour proliferation‐promoting effects by miR‐30e rescued by *PTPN13*

**Figure 5:**
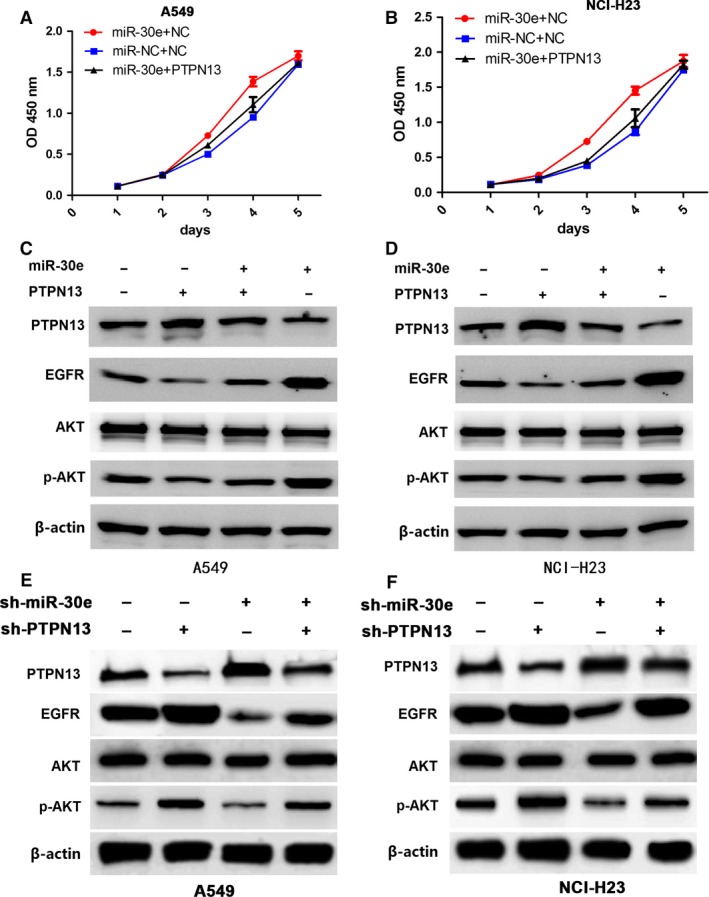
*PTPN13* rescued tumour proliferation‐promoting effects by miR‐30e. (**A** and **B**) The effects of *PTPN13* overexpression on cell proliferation in miR‐30e‐transfected A549 and NCI‐H23 cells indicated by MTT assay. (**C** and **D**) The effects of *PTPN13* overexpression on the protein expression of EGFR/AKT pathway in miR‐30e‐transfected A549 and NCI‐H23 cells indicated by western blotting. (**E** and **F**) The effects of *PTPN13* knockdown on the protein expression of EGFR/AKT pathway in sh‐miR‐30e‐transfected A549 and NCI‐H23 cells indicated by Western blotting.

To gain insight into the molecular mechanisms by which *PTPN13* rescued miR‐30e‐caused cell proliferation, the *PTPN13* expression vector constructed to restore *PTPN13* expression was co‐transfected with miR‐30e into A549 and NCI‐H23 cells. The results showed that *PTPN13* rescued the miR‐30e‐promoted cell proliferation (Fig. 5A and B). Additionally, enforced expression of *PTPN13* could rescue miR‐30e‐induced upregulation of EGFR/AKT pathway as downstream regulation factors of *PTPN13* (Fig. 5C and D). Also,we found that knockdown of *PTPN13* could rescue sh‐miR‐30e‐induced down‐regulation of EGFR/AKT pathway as downstream regulation factors of *PTPN13* (Fig. 5E and F).

### A549 xenograft growth promoted by miR‐30e

**Figure 6:**
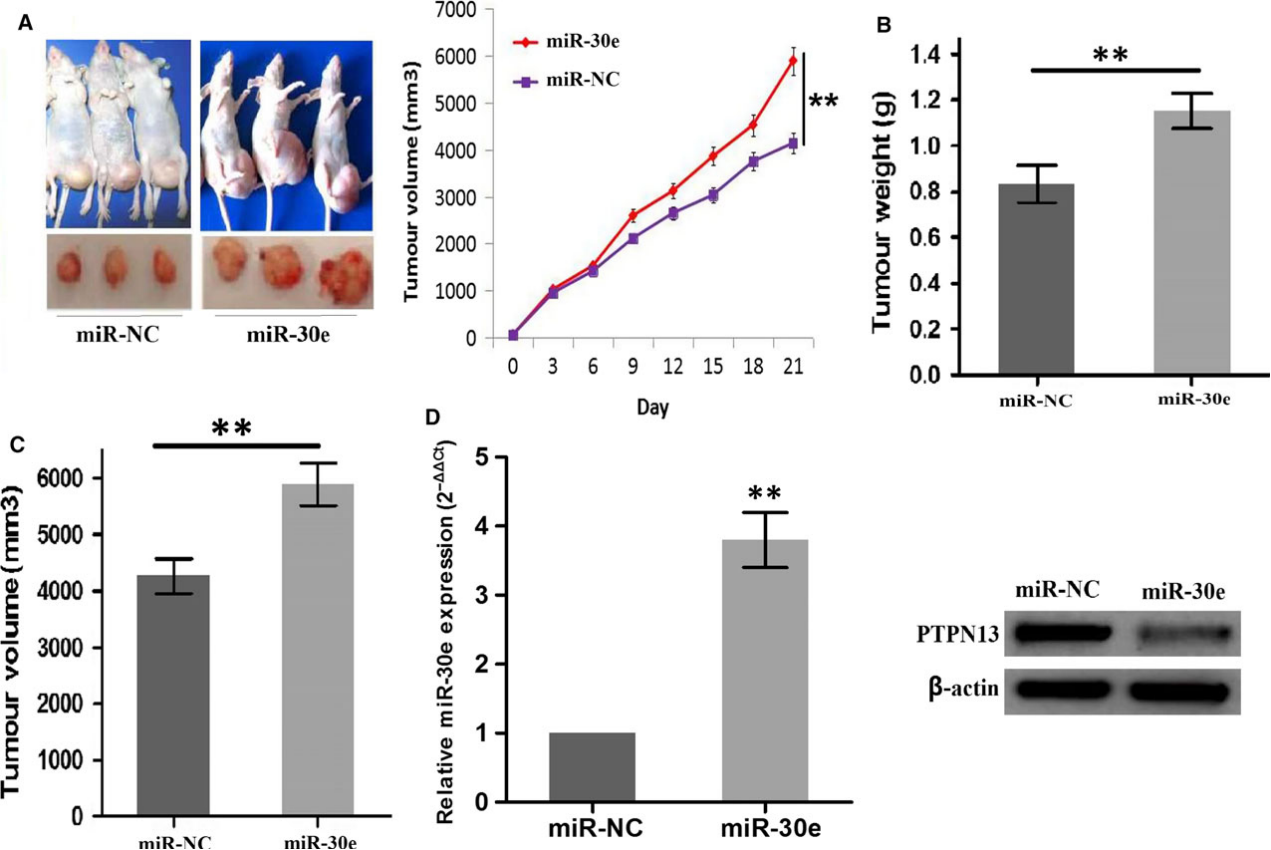
miR‐30e overexpression inhibited A549 xenograft tumour growth. (**A**) The effects of miR‐30e overexpression on proliferation activity of A549 xenograft tumours. (**B** and **C**) The effects of miR‐30e overexpression on the average weight and volumes in xenograft tumours. (**D**) The expression level of miR‐30e and PTPN13 in LAC tissues from miR‐NC and miR‐30e groups. **P < 0.01.

Having investigated that miR‐30e enhanced the LAC growth *in vitro,* we further conformed the promoting effects of miR‐30e on LAC *in vivo*. A subcutaneous A549 xenograft tumour model was established to observe tumour growth activity affected by miR‐30e. During the tumour growth period, it was found that the proliferation rates of tumours were significantly increased in miR‐30e group compared to miR‐NC group (Fig. 6A). When the tumours were harvested, the average weight and volumes were elevated in miR‐30e group in comparison with miR‐NC group (**P < 0.01, Fig. 6B and C). The miR‐30e expression was increased (**P < 0.01), while the PTPN13 protein expression was decreased in miR‐30e group compared with miR‐NC group (Fig. 6D).

## Discussion

Lung cancer is still a major health problem for human owing to the high incidence rate and mortality rate. Tumour‐related molecular abnormalities play a key role in lung cancer progression and therapy 16. Some miRNAs such as miR‐182, miR‐483, and miR‐224 promotes LAC proliferation, invasion and metastasis 17, 18, 19, while miR‐361, miR‐340 and miR‐33b inhibits LAC cell growth and invasion *via* regulation of their target genes *SH2B1, p27* and Wnt/β‐catenin/ZEB1 signalling 20, 21, 22. A previous study uncovered that miR‐30e enhanced glioma cell invasion through EGFR stabilization by directly targeting *CBL‐B* 23. Consistent with the reports, miR‐30e has a well‐characterized association with cancer progression 13. Although miR‐30e‐5p was down‐regulated in NSCLC tissues, high expression of miR‐30e‐5p was found to be associated with shorter OS in NSCLC patients 14. NSCLC tissues included LAC and other types, which might have a different expression level of miR‐30e in these tissues. Thus, we found that miR‐30e expression was significantly up‐regulated in LAC tissues, and positively associated with the tumour size and represented an independent unfavourable prognosis for OS and recurrence in LAC patients, suggesting that miR‐30e might have the potential as a biomarker for survival and recurrence of LAC patients.

Functionally, miR‐30e accelerated human glioma cell invasiveness by targeting CBL‐B or dilapidating the NF‐κB/IκBα negative feedback loop 23, 24. MiR‐30e‐induced apoptosis and benefited imatinib treatment in chronic myeloid leukaemia *via* inhibition of BCR‐ABL 25 and repressed proliferation of hepatocellular carcinoma cells *via* targeting P4HA1 26. Thus, a detailed understanding of how miR‐30e functions in LAC cells is required. We found that miR‐30e overexpression facilitated cell proliferation and colony formation, while miR‐30e knockdown inhibited cell proliferation in LAC cells, indicating that miR‐30e might act as an oncogene in LAC.

Tyrosine phosphatase gene superfamily including *PTPN13* is implicated in signalling pathways underlying tumorigenesis 27. *PTPN13* restrained anchorage‐independent growth and tumorigenicity in lung cancer *via* inactivation of the EGFR and *HER2* 28, participated in the lung cancer invasion and negatively correlated with cancer grade and stage 29. *PTPN13* as tumour suppressor gene induced cell apoptosis *via* inhibition of the IRS‐1/PI3K/Akt signalling pathway 30. Our present studies indicated that *PTPN13* was identified as a direct target of miR‐30e, and enhanced expression of *PTPN13* decreased cell proliferation and colony formation, and rescued the proliferation‐promoting effects of miR‐30e through inhibition of the EGFR//AKT signalling, indicating that miR‐30e might promote LAC growth by targeting *PTPN13*.

In conclusion, our findings showed that imiR‐30e was up‐regulated in LAC, positively associated with tumour size and represented an independent prognostic factor for survival and recurrence of LAC patients. Enforced expression of miR‐30e promoted LAC growth by targeting *PTPN13*. These data suggested that miR‐30e may function as an oncogene in LAC and serve as a potential therapeutic target for LAC.

## Conflict of interest

The authors declare no conflict of interest.

## Supporting information


**Figure S1** miR‐30e overexpression promoted cell proliferation.

**Table S1** The clinicopathologic data of LAC patients.
**Table S2** The correlation of miR‐30e expression with clinicopathologic characteristics of LAC patients shown by TCGA.
**Table S3** Univariate and multivariate analyses of factors associated with overall survival.
**Table S4** Univariate and multivariate analyses of factors associated with cumulative recurrence.
**Table S5** The predicted target genes of miR‐30e in cancer tissues.
