# Supplementary figures and images for: MicroRNA‐30e‐5p promotes cell growth by targeting PTPN13 and indicates poor survival and recurrence in lung adenocarcinoma
Source: J Cell Mol Med. 2017 Jun 27;21(11):2852–62. doi: 10.1111/jcmm.13198 (PMC5661247; doi:10.1111/jcmm.13198)

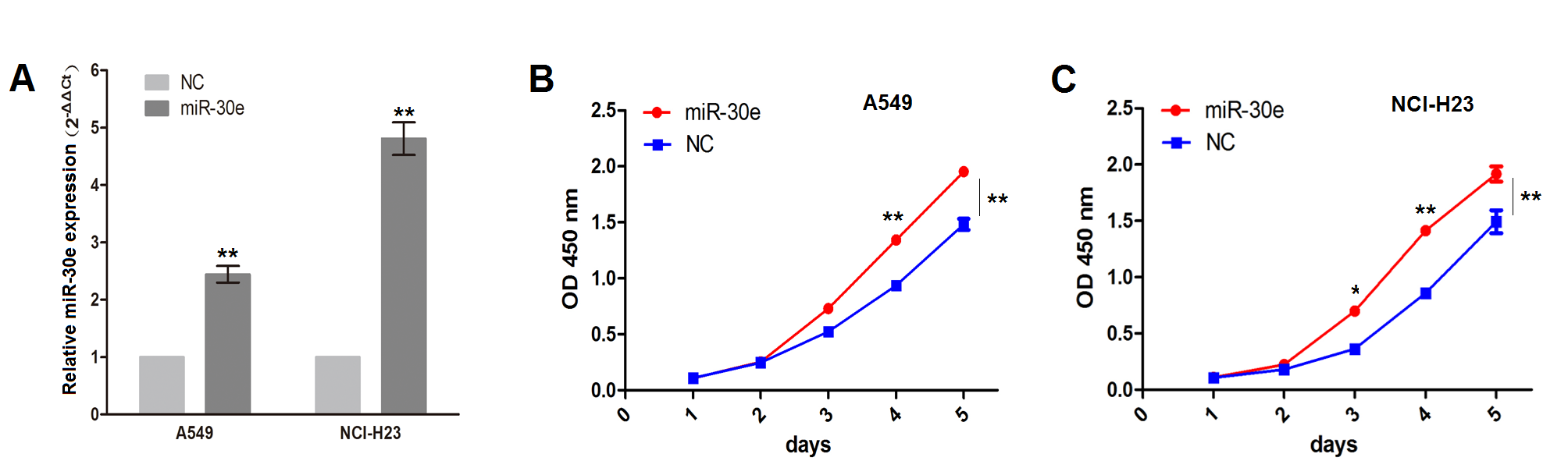

Supplement: Supplementary file 1 — Figure S1 miR‐30e overexpression promoted cell proliferation. [file JCMM-21-2852-s001.tif]
