# Supplementary material for: MicroRNA‐30e‐5p promotes cell growth by targeting PTPN13 and indicates poor survival and recurrence in lung adenocarcinoma
Source: J Cell Mol Med. 2017 Jun 27;21(11):2852–62. doi: 10.1111/jcmm.13198 (PMC5661247; doi:10.1111/jcmm.13198)
Supplement: Supplementary file 2 — Table S1 The clinicopathologic data of LAC patients. Table S2 The correlation of miR‐30e expression with clinicopathologic characteristics of LAC patients shown by TCGA. Table S3 Univariate and multivariate analyses of factors associated with overall survival. Table S4 Univariate and multivariate analyses of factors associated with cumulative recurrence. Table S5 The predicted target genes of miR‐30e in cancer tissues. [file JCMM-21-2852-s002.docx]

Table S1 The clinicopathologic data of LAC patients

| Variables | Number of cases (%) |
| --- | --- |
| Number of patients | 78 (100%) |
| *Age (years)* |  |
| ≤60 | 32 (41.03%) |
| ＞60 | 46 (58.97%) |
| *Sex* |  |
| Male | 51 (65.38%) |
| Female | 27 (34.62%) |
| Smoking status |  |
| Current smokers | 12 (15.38%) |
| Previous smokers | 45 (57.69%) |
| Slight/No smokers | 21 (26.93%) |
| *Tumor size (cm)* |  |
| ≤ 3. | 42 (53.85%) |
| ＞3 | 36 (46.15%) |
| *Adenocarcinoma subtypes* |  |
| Adenocarcinoma in situ (AIS) | 3 (3.85%) |
| Minimally invasive adenocarcinoma (MIA) | 7 (8.97%) |
| Invasive adenocarcinoma (IA) |  |
| Lepidic predominant | 37(47.44%) |
| Acinar predominant | 14(17.85%) |
| Papillary predominant | 11(14.10%) |
| *Variants of invasive adenocarcinoma§* |  |
| Invasive mucinous adenocarcinoma (IMA) | 6(7.69%) |
| *Pathological stage* |  |
| Ⅰ/Ⅱ | 57 (73.07%) |
| Ⅲ/Ⅳ | 21 (26.93%) |
| *pT stage* |  |
| T1/T2 | 66 (84.62%) |
| T3/T4 | 12 (15.38%) |
| *pN stage* |  |
| Negative | 43 (55.13%) |
| Positive | 35 (44.87%) |
| *pM* |  |
| Negative | 69(88.46%) |
| Positive | 9 (11.54%) |

Table S2 The correlation of miR-30e expression with clinicopathologic

characteristics of LAC patients shown by TCGA

| Variables | Cases  (n) | miR-30e | | *P* value |
| --- | --- | --- | --- | --- |
|  |  | High | Low |  |
| Total | 227 | 92 | 135 |  |
| *Age (years)* |  |  |  |  |
| ≥60 | 162 | 68 | 94 |  |
| <60 | 65 | 24 | 41 | 0.484 |
| *Sex* |  |  |  |  |
| Male | 100 | 36 | 64 |  |
| Female | 127 | 56 | 71 | 0.219 |
| *Smoking status* |  |  |  |  |
| Current smokers | 37 | 19 | 18 |  |
| Previous smokers | 133 | 52 | 81 | 0.329 |
| Slight/No | 57 | 21 | 36 |  |
| *Tumor size (cm)* |  |  |  |  |
| ≥2 | 16 | 9 | 7 |  |
| <2 | 211 | 83 | 128 | 0.185 |
| *Adenocarcinoma subtypes* |  |  |  |  |
| Adenocarcinoma mix subtype | 57 | 22 | 35 |  |
| Lung acinar adenocarcinoma | 10 | 4 | 6 |  |
| Lung Bronchioloalveolar Carcinoma  Lung Papillary Adenocarcinoma | 18  10 | 8  7 | 10  3 | 0.408 |
| Not otherwise specified | 132 | 51 | 81 |  |
| *Pathological stage* |  |  |  |  |
| Ⅰ/Ⅱ | 173 | 75 | 98 |  |
| Ⅲ/Ⅳ | 54 | 17 | 37 | 0.122 |
| *pT stage* |  |  |  |  |
| T1/T2 | 198 | 85 | 113 |  |
| T3/T4 | 29 | 7 | 22 | 0.055 |
| *pN stage* |  |  |  |  |
| Positive | 84 | 28 | 56 |  |
| Negative | 143 | 64 | 79 | 0.091 |
| *pM* |  |  |  |  |
| Positive | 8 | 4 | 4 |  |
| Negative | 219 | 88 | 131 | 0.579 |

Table S3 Univariate and multivariate analyses of factors associated with

overall survival

|  | OS | | | |
| --- | --- | --- | --- | --- |
|  |  | Multivariate | | |
| Factors | Univariate*P* | HR | 95% CI | *P* value |
| Age (years) (≥60 vs. <60) | 0.417 |  |  | NA |
| Sex (male vs. female) | 0.275 |  |  | NA |
| Smoking status  (Current vs Previous vs. Slight/No) | 0.216 |  |  | NA |
| Tumor size (cm) (≥3 vs. <3) | 0.081 |  |  | NS |
| Adenocarcinoma subtypes  (AIS vs. MIA vs. IA vs IMA) | 0.017 | 1.237 | 0.987-2.136 | 0.035 |
| Pathological staging (III/IV vs. I/II.) | 0.024 | 1.764 | 1.015-2.683 | 0.037 |
| pT (negative vs. Positive) | 0.127 |  |  | NA |
| pN (negative vs. Positive) | 0.072 |  |  | NA |
| pM (negative vs. Positive) | 0.035 | 1.219 | 0.914-1.883 | 0.120 |
| miR-30e expression (high vs. low) | 0.017 | 1.842 | 1.397-2.654 | 0.024 |

Table S4 Univariate and multivariate analyses of factors associated with cumulative recurrence

|  | Recurrence | | | |
| --- | --- | --- | --- | --- |
|  |  | Multivariate | | |
| Factors | Univariate,  *P* | HR | 95% CI | *P* value |
| Age (years) (≥60 vs. <60) | 0.389 |  |  | NA |
| Sex (male vs. female) | 0.316 |  |  | NA |
| Smoking status  (Current vs Previous vs. Slight/No) | 0.179 |  |  | NA |
| Tumor size (cm) (≥3 vs. <5) | 0.095 |  |  | NS |
| Adenocarcinoma subtypes  (AIS vs. MIA vs. IA vs IMA) | 0.013 | 0.928 | 0.795-1.269 | 0.027 |
| Pathological staging (III/IV vs. I/II.) | 0.016 | 1.275 | 0.981-2.634 | 0.031 |
| pT (negative vs. Positive) | 0.116 |  |  | NA |
| pN (negative vs. Positive) | 0.098 |  |  | NA |
| pM (negative vs. Positive) | 0.023 | 1.241 | 0.895-1.852 | 0.041 |
| miR-30e expression (high vs. low) | 0.021 | 1.726 | 1.131-2.497 | 0.036 |

Table S5 The predicted target genes of miR-30e in cancer tissues

| name | geneName | position | targetScanSites | picTarSites | RNA22Sites | PITASites | miRandaSites | programNum | CancerNum |
| --- | --- | --- | --- | --- | --- | --- | --- | --- | --- |
| hsa-miR-30e-5p | SEC24A | chr5:134062121-134062128[+] | 108[7] | 0[0] | 0[0] | 108[7] | 108[7] | 3 | 3 |
| hsa-miR-30e-5p | PTPN13 | chr4:87736164-87736170[+] | 218[8] | 0[0] | 0[0] | 218[8] | 218[8] | 3 | 3 |
| hsa-miR-30e-5p | PTPN13 | chr4:87736164-87736170[+] | 218[8] | 0[0] | 0[0] | 218[8] | 218[8] | 3 | 3 |
| hsa-miR-30e-5p | DPY19L1 | chr7:34968534-34968541[-] | 260[5] | 0[0] | 0[0] | 260[5] | 260[5] | 3 | 7 |
| hsa-miR-30e-5p | HERC2 | chr15:28356649-28356655[-] | 122[2] | 0[0] | 0[0] | 122[2] | 139[5] | 3 | 4 |
| hsa-miR-30e-5p | JARID2 | chr6:15522197-15522203[+] | 83[6] | 83[6] | 0[0] | 83[6] | 83[6] | 4 | 4 |
| hsa-miR-30e-5p | ZBTB7A | chr19:4046483-4046490[-] | 1776[15] | 0[0] | 0[0] | 1776[15] | 1836[21] | 3 | 7 |
| hsa-miR-30e-5p | TANK | chr2:162092282-162092289[+] | 146[7] | 0[0] | 0[0] | 146[7] | 146[7] | 3 | 4 |
| hsa-miR-30e-5p | C14orf129 | chr14:96852859-96852866[+] | 54[7] | 0[0] | 0[0] | 54[7] | 68[7] | 3 | 5 |
| hsa-miR-30e-5p | GNAI2 | chr3:50296180-50296187[+] | 643[13] | 643[13] | 0[0] | 643[13] | 643[13] | 4 | 12 |
| hsa-miR-30e-5p | GNAI2 | chr3:50296457-50296464[+] | 83[10] | 78[7] | 0[0] | 83[10] | 83[10] | 4 | 12 |
| hsa-miR-30e-5p | FAM104A | chr17:71205512-71205518[-] | 652[15] | 0[0] | 0[0] | 652[15] | 652[15] | 3 | 5 |
| hsa-miR-30e-5p | NFIB | chr9:14082652-14082658[-] | 1098[8] | 1098[8] | 0[0] | 1098[8] | 2196[8] | 4 | 3 |
| hsa-miR-30e-5p | NFIB | chr9:14087828-14087834[-] | 329[6] | 329[6] | 0[0] | 329[6] | 723[8] | 4 | 3 |
| hsa-miR-30e-5p | UBE2V2 | chr8:48973994-48974001[+] | 454[6] | 0[0] | 0[0] | 444[5] | 539[10] | 3 | 5 |
| hsa-miR-30e-5p | JOSD1 | chr22:39081671-39081678[-] | 395[13] | 0[0] | 0[0] | 395[13] | 395[13] | 3 | 6 |
| hsa-miR-30e-5p | LMBR1L | chr12:49491142-49491149[-] | 247[11] | 0[0] | 0[0] | 266[11] | 587[16] | 3 | 3 |
| hsa-miR-30e-5p | LMBR1L | chr12:49491193-49491200[-] | 779[16] | 0[0] | 0[0] | 779[16] | 779[16] | 3 | 3 |
| hsa-miR-30e-5p | CCDC43 | chr17:42756103-42756110[-] | 506[13] | 0[0] | 0[0] | 253[13] | 506[13] | 3 | 4 |
| hsa-miR-30e-5p | VAMP3 | chr1:7840267-7840274[+] | 35[4] | 0[0] | 0[0] | 35[4] | 35[6] | 3 | 5 |
| hsa-miR-30e-5p | MYBL2 | chr20:42344758-42344765[+] | 2336[11] | 0[0] | 0[0] | 2336[11] | 2336[11] | 3 | 5 |
| hsa-miR-30e-5p | ATP2A2 | chr12:110784901-110784908[+] | 1442[6] | 1442[6] | 0[0] | 1442[6] | 0[0] | 3 | 3 |
| hsa-miR-30e-5p | MAP3K12 | chr12:53874561-53874568[-] | 65[7] | 0[0] | 0[0] | 65[7] | 65[7] | 3 | 5 |
| hsa-miR-30e-5p | CFL2 | chr14:35181369-35181375[-] | 731[15] | 731[15] | 0[0] | 731[15] | 731[15] | 4 | 6 |
| hsa-miR-30e-5p | PON2 | chr7:95034217-95034223[-] | 93[7] | 93[7] | 0[0] | 0[0] | 1802[8] | 3 | 4 |
| hsa-miR-30e-5p | EIF2C1 | chr1:36386824-36386831[+] | 0[6] | 0[0] | 0[0] | 0[6] | 466[12] | 3 | 3 |
| hsa-miR-30e-5p | PIP4K2B | chr17:36923751-36923758[-] | 112[14] | 112[14] | 0[0] | 0[0] | 112[14] | 3 | 5 |
| hsa-miR-30e-5p | TUSC3 | chr8:15621755-15621761[+] | 842[6] | 0[0] | 0[0] | 421[6] | 2526[6] | 3 | 6 |
| hsa-miR-30e-5p | ZCCHC14 | chr16:87441995-87442001[-] | 1196[7] | 0[0] | 0[0] | 1196[7] | 1196[7] | 3 | 4 |
| hsa-miR-30e-5p | NR4A2 | chr2:157180970-157180977[-] | 402[7] | 0[0] | 0[0] | 402[7] | 402[7] | 3 | 3 |
| hsa-miR-30e-5p | SLC6A9 | chr1:44463041-44463048[-] | 576[8] | 576[8] | 0[0] | 576[8] | 576[8] | 4 | 3 |
| hsa-miR-30e-5p | ELL2 | chr5:95223171-95223178[-] | 60[5] | 0[0] | 0[0] | 60[5] | 60[5] | 3 | 7 |
| hsa-miR-30e-5p | KRAS | chr12:25358477-25358483[-] | 127[9] | 127[9] | 0[0] | 127[9] | 321[9] | 4 | 4 |
| hsa-miR-30e-5p | RAP2B | chr3:152881807-152881813[+] | 1021[12] | 0[0] | 0[0] | 1021[12] | 1024[15] | 3 | 6 |
| hsa-miR-30e-5p | CSNK1A1 | chr5:148875579-148875586[-] | 390[9] | 0[0] | 0[0] | 390[9] | 780[9] | 3 | 7 |
| hsa-miR-30e-5p | SEMA3A | chr7:83590576-83590583[-] | 48[2] | 0[0] | 0[0] | 48[2] | 69[5] | 3 | 9 |
| hsa-miR-30e-5p | DHX40 | chr17:57685522-57685528[+] | 357[7] | 0[0] | 0[0] | 357[7] | 376[9] | 3 | 3 |
| hsa-miR-30e-5p | VAPA | chr18:9954267-9954273[+] | 609[11] | 0[0] | 0[0] | 609[11] | 609[11] | 3 | 4 |
| hsa-miR-30e-5p | AHNAK | chr11:62283922-62283928[-] | 5516[12] | 0[0] | 0[0] | 5516[12] | 5516[14] | 3 | 7 |
| hsa-miR-30e-5p | SHOC2 | chr10:112772051-112772058[+] | 71[6] | 0[0] | 0[0] | 71[6] | 71[6] | 3 | 4 |
| hsa-miR-30e-5p | SH3PXD2A | chr10:105353838-105353845[-] | 175[5] | 0[0] | 0[0] | 175[5] | 175[5] | 3 | 11 |
| hsa-miR-30e-5p | ADAM9 | chr8:38962166-38962173[+] | 2161[4] | 0[0] | 0[0] | 2161[4] | 10855[5] | 3 | 7 |
| hsa-miR-30e-5p | TMEM87A | chr15:42503272-42503279[-] | 1694[5] | 0[0] | 0[0] | 1694[5] | 1694[5] | 3 | 4 |
| hsa-miR-30e-5p | YPEL5 | chr2:30381946-30381953[+] | 93[5] | 93[5] | 0[0] | 93[5] | 187[5] | 4 | 4 |
| hsa-miR-30e-5p | GALNT7 | chr4:174243053-174243060[+] | 719[4] | 0[0] | 0[0] | 719[4] | 762[9] | 3 | 4 |
| hsa-miR-30e-5p | AP2A1 | chr19:50310185-50310192[+] | 531[18] | 0[0] | 0[0] | 531[18] | 537[18] | 3 | 8 |
| hsa-miR-30e-5p | FAM160B1 | chr10:116622255-116622262[+] | 994[12] | 994[12] | 0[0] | 0[0] | 994[14] | 3 | 5 |
| hsa-miR-30e-5p | UBE2I | chr16:1374846-1374853[+] | 29[4] | 29[4] | 0[0] | 24[3] | 76[5] | 4 | 4 |
| hsa-miR-30e-5p | ZDHHC17 | chr12:77245988-77245995[+] | 157[12] | 0[0] | 0[0] | 157[12] | 157[12] | 3 | 3 |
| hsa-miR-30e-5p | FAM91A1 | chr8:124827650-124827657[+] | 1002[17] | 1002[17] | 0[0] | 1002[17] | 1002[17] | 4 | 4 |
| hsa-miR-30e-5p | MAML1 | chr5:179203992-179203999[+] | 550[8] | 0[0] | 0[0] | 550[8] | 550[8] | 3 | 3 |
| hsa-miR-30e-5p | NEFL | chr8:24810035-24810042[-] | 2302[10] | 0[0] | 0[0] | 2302[10] | 2302[10] | 3 | 7 |
| hsa-miR-30e-5p | SMARCD2 | chr17:61909975-61909982[-] | 39[12] | 0[0] | 0[0] | 39[12] | 39[12] | 3 | 4 |
| hsa-miR-30e-5p | RAP1B | chr12:69053728-69053735[+] | 232[5] | 0[0] | 0[0] | 232[5] | 384[10] | 3 | 8 |
| hsa-miR-30e-5p | SLC4A7 | chr3:27415651-27415658[-] | 860[9] | 0[0] | 0[0] | 860[9] | 860[9] | 3 | 4 |
| hsa-miR-30e-5p | FBXO45 | chr3:196312287-196312294[+] | 1365[20] | 0[0] | 0[0] | 1365[20] | 1365[20] | 3 | 7 |
| hsa-miR-30e-5p | RHOB | chr2:20649125-20649132[+] | 5667[9] | 0[0] | 0[0] | 5667[9] | 5677[10] | 3 | 7 |
| hsa-miR-30e-5p | ERLIN1 | chr10:101911538-101911545[-] | 1228[15] | 0[0] | 0[0] | 1228[15] | 1228[15] | 3 | 4 |
| hsa-miR-30e-5p | YWHAZ | chr8:101932830-101932836[-] | 241[5] | 241[5] | 0[0] | 241[5] | 252[6] | 4 | 9 |
| hsa-miR-30e-5p | AFF4 | chr5:132215289-132215296[-] | 374[10] | 374[10] | 0[0] | 374[10] | 450[11] | 4 | 4 |
| hsa-miR-30e-5p | TAOK1 | chr17:27870195-27870201[+] | 216[14] | 0[0] | 0[0] | 216[14] | 217[14] | 3 | 4 |
| hsa-miR-30e-5p | TAOK1 | chr17:27871195-27871202[+] | 60[5] | 0[0] | 0[0] | 60[5] | 121[6] | 3 | 4 |
| hsa-miR-30e-5p | QKI | chr6:163990949-163990956[+] | 219[5] | 0[0] | 0[0] | 219[5] | 1463[11] | 3 | 5 |
| hsa-miR-30e-5p | NEDD4 | chr15:56121128-56121135[-] | 591[7] | 591[7] | 0[0] | 591[7] | 591[7] | 4 | 8 |
| hsa-miR-30e-5p | NEDD4 | chr15:56121146-56121153[-] | 591[7] | 591[7] | 0[0] | 591[7] | 591[7] | 4 | 8 |
| hsa-miR-30e-5p | PRDM1 | chr6:106557731-106557738[+] | 2058[11] | 0[0] | 0[0] | 1029[11] | 1029[11] | 3 | 4 |
| hsa-miR-30e-5p | B4GALT6 | chr18:29205441-29205448[-] | 7[5] | 0[0] | 7[5] | 7[5] | 7[5] | 4 | 4 |
| hsa-miR-30e-5p | SOCS3 | chr17:76353067-76353074[-] | 697[7] | 0[0] | 0[0] | 697[7] | 697[7] | 3 | 8 |
| hsa-miR-30e-5p | SLC38A2 | chr12:46753602-46753609[-] | 5033[10] | 0[0] | 0[0] | 5033[10] | 5041[10] | 3 | 8 |
| hsa-miR-30e-5p | ADAM19 | chr5:156904411-156904418[-] | 142[6] | 0[0] | 0[0] | 142[6] | 284[6] | 3 | 9 |
| hsa-miR-30e-5p | SOX4 | chr6:21598054-21598061[+] | 1147[18] | 0[0] | 0[0] | 1147[18] | 1147[18] | 3 | 3 |
| hsa-miR-30e-5p | RAB23 | chr6:57054105-57054112[-] | 593[11] | 0[0] | 0[0] | 593[11] | 593[11] | 3 | 9 |
| hsa-miR-30e-5p | FNDC3A | chr13:49783193-49783200[+] | 1813[6] | 0[0] | 0[0] | 1813[6] | 3627[6] | 3 | 3 |
| hsa-miR-30e-5p | SATB2 | chr2:200135164-200135170[-] | 51[3] | 0[0] | 0[0] | 51[3] | 61[7] | 3 | 8 |
| hsa-miR-30e-5p | GNPDA1 | chr5:141380274-141380281[-] | 306[13] | 0[0] | 320[14] | 306[13] | 320[14] | 4 | 4 |
| hsa-miR-30e-5p | MYH10 | chr17:8377592-8377598[-] | 38[5] | 0[0] | 0[0] | 38[5] | 38[5] | 3 | 9 |
| hsa-miR-30e-5p | STK39 | chr2:168810593-168810600[-] | 299[6] | 0[0] | 0[0] | 299[6] | 299[6] | 3 | 6 |
| hsa-miR-30e-5p | DDIT4 | chr10:74035687-74035694[+] | 4488[18] | 0[0] | 0[0] | 4488[18] | 4503[19] | 3 | 8 |
| hsa-miR-30e-5p | PPP1R12A | chr12:80169423-80169430[-] | 910[9] | 910[9] | 0[0] | 910[9] | 910[9] | 4 | 4 |
| hsa-miR-30e-5p | MAP3K5 | chr6:136878773-136878779[-] | 867[7] | 0[0] | 0[0] | 867[7] | 867[7] | 3 | 3 |
| hsa-miR-30e-5p | STIM2 | chr4:27024989-27024995[+] | 320[9] | 0[0] | 0[0] | 160[9] | 481[9] | 3 | 3 |
| hsa-miR-30e-5p | RHEBL1 | chr12:49458698-49458705[-] | 386[8] | 0[0] | 0[0] | 386[8] | 386[8] | 3 | 5 |
| hsa-miR-30e-5p | REEP3 | chr10:65381230-65381236[+] | 3519[20] | 0[0] | 0[0] | 3519[20] | 4180[24] | 3 | 5 |
| hsa-miR-30e-5p | EXTL2 | chr1:101339239-101339246[-] | 303[11] | 0[0] | 0[0] | 303[11] | 304[11] | 3 | 3 |
| hsa-miR-30e-5p | AMOTL2 | chr3:134074994-134075001[-] | 6838[8] | 0[0] | 0[0] | 6838[8] | 6838[8] | 3 | 4 |
| hsa-miR-30e-5p | JDP2 | chr14:75936817-75936824[+] | 519[4] | 519[4] | 0[0] | 519[4] | 525[5] | 4 | 5 |
| hsa-miR-30e-5p | RBM12 | chr20:34240292-34240299[-] | 119[6] | 0[0] | 0[0] | 119[6] | 137[8] | 3 | 5 |
| hsa-miR-30e-5p | GRB10 | chr7:50660276-50660282[-] | 87[3] | 0[0] | 0[0] | 87[3] | 133[8] | 3 | 5 |
| hsa-miR-30e-5p | JAK1 | chr1:65298944-65298950[-] | 7265[21] | 0[0] | 0[0] | 7265[21] | 7275[22] | 3 | 6 |
| hsa-miR-30e-5p | CALU | chr7:128411517-128411524[+] | 3184[5] | 3184[5] | 0[0] | 3184[5] | 3184[5] | 4 | 9 |
| hsa-miR-30e-5p | C12orf76 | chr12:110479317-110479324[-] | 114[7] | 0[0] | 114[7] | 0[0] | 228[7] | 3 | 3 |
| hsa-miR-30e-5p | DLG5 | chr10:79551842-79551848[-] | 290[5] | 0[0] | 0[0] | 290[5] | 295[6] | 3 | 3 |
| hsa-miR-30e-5p | ELOVL5 | chr6:53132400-53132407[-] | 897[10] | 0[0] | 0[0] | 897[10] | 1793[12] | 3 | 3 |
| hsa-miR-30e-5p | UBE2J1 | chr6:90039317-90039324[-] | 53[9] | 0[0] | 0[0] | 53[9] | 59[10] | 3 | 4 |
| hsa-miR-30e-5p | ELL | chr19:18553629-18553636[-] | 78[5] | 0[0] | 0[0] | 78[5] | 135[8] | 3 | 3 |
| hsa-miR-30e-5p | CHD1 | chr5:98191614-98191621[-] | 1728[8] | 0[0] | 0[0] | 1728[8] | 1728[8] | 3 | 4 |
| hsa-miR-30e-5p | CHD1 | chr5:98191826-98191832[-] | 1413[20] | 0[0] | 0[0] | 1413[20] | 1413[20] | 3 | 4 |
| hsa-miR-30e-5p | KIAA0355 | chr19:34845805-34845812[+] | 287[8] | 0[0] | 0[0] | 287[8] | 303[9] | 3 | 4 |
| hsa-miR-30e-5p | SEC23A | chr14:39501384-39501391[-] | 675[6] | 0[0] | 0[0] | 675[6] | 698[8] | 3 | 12 |
| hsa-miR-30e-5p | UBE3C | chr7:157060902-157060909[+] | 181[13] | 181[13] | 0[0] | 181[13] | 181[13] | 4 | 4 |
| hsa-miR-30e-5p | EAF1 | chr3:15484023-15484030[+] | 87[5] | 87[5] | 0[0] | 87[5] | 102[5] | 4 | 3 |
| hsa-miR-30e-5p | PPP3R1 | chr2:68406481-68406488[-] | 1071[15] | 0[0] | 0[0] | 1071[15] | 1071[15] | 3 | 6 |
| hsa-miR-30e-5p | TMCC1 | chr3:129370202-129370209[-] | 110[7] | 0[0] | 110[7] | 110[7] | 110[7] | 4 | 3 |
| hsa-miR-30e-5p | MAP4K4 | chr2:102510839-102510846[+] | 0[4] | 0[4] | 0[0] | 0[4] | 24[6] | 4 | 8 |
| hsa-miR-30e-5p | ASCC3 | chr6:100956929-100956936[-] | 68[6] | 0[0] | 0[0] | 68[6] | 80[8] | 3 | 3 |
| hsa-miR-30e-5p | WDR82 | chr3:52290591-52290598[-] | 67[7] | 0[0] | 67[7] | 0[0] | 67[7] | 3 | 5 |
| hsa-miR-30e-5p | GFPT2 | chr5:179728340-179728347[-] | 420[7] | 0[0] | 0[0] | 420[7] | 420[7] | 3 | 12 |
| hsa-miR-30e-5p | RARG | chr12:53604425-53604432[-] | 32[4] | 0[0] | 0[0] | 32[4] | 191[5] | 3 | 4 |
| hsa-miR-30e-5p | LMBR1 | chr7:156473673-156473680[-] | 13[7] | 0[0] | 0[0] | 13[7] | 49[11] | 3 | 5 |
| hsa-miR-30e-5p | METAP2 | chr12:95909529-95909536[+] | 495[5] | 0[0] | 0[0] | 495[5] | 989[5] | 3 | 4 |
| hsa-miR-30e-5p | PCDH10 | chr4:134111567-134111574[+] | 328[7] | 0[0] | 0[0] | 328[7] | 328[7] | 3 | 6 |
| hsa-miR-30e-5p | TBC1D10B | chr16:30368495-30368502[-] | 317[15] | 0[0] | 0[0] | 317[15] | 317[15] | 3 | 4 |
| hsa-miR-30e-5p | GLCE | chr15:69563897-69563903[+] | 50[6] | 0[0] | 0[0] | 50[6] | 50[6] | 3 | 4 |
| hsa-miR-30e-5p | GLCE | chr15:69564330-69564337[+] | 145[6] | 0[0] | 0[0] | 145[6] | 176[9] | 3 | 4 |
| hsa-miR-30e-5p | CAMK2D | chr4:114375491-114375498[-] | 225[6] | 75[6] | 0[0] | 75[6] | 225[6] | 4 | 3 |
| hsa-miR-30e-5p | PER2 | chr2:239153081-239153087[-] | 648[9] | 0[0] | 0[0] | 648[9] | 666[9] | 3 | 4 |
| hsa-miR-30e-5p | ARL15 | chr5:53182163-53182169[-] | 122[8] | 0[0] | 0[0] | 122[8] | 122[8] | 3 | 6 |
| hsa-miR-30e-5p | SH3RF1 | chr4:170017373-170017380[-] | 156[9] | 0[0] | 0[0] | 156[9] | 156[9] | 3 | 3 |
| hsa-miR-30e-5p | RASA1 | chr5:86687400-86687407[+] | 442[10] | 0[0] | 0[0] | 442[10] | 884[10] | 3 | 4 |
| hsa-miR-30e-5p | LRCH2 | chrX:114347205-114347212[-] | 6[5] | 0[0] | 0[0] | 6[5] | 35[8] | 3 | 6 |
| hsa-miR-30e-5p | PGM2L1 | chr11:74047054-74047060[-] | 63[5] | 0[0] | 0[0] | 63[5] | 63[5] | 3 | 6 |
| hsa-miR-30e-5p | ZFAND5 | chr9:74970109-74970116[-] | 145[13] | 0[0] | 0[0] | 145[13] | 145[13] | 3 | 3 |
| hsa-miR-30e-5p | GMEB2 | chr20:62221167-62221174[-] | 356[11] | 356[11] | 0[0] | 356[11] | 356[11] | 4 | 3 |
| hsa-miR-30e-5p | SCAMP1 | chr5:77774094-77774100[+] | 228[5] | 0[0] | 0[0] | 228[5] | 239[9] | 3 | 3 |
| hsa-miR-30e-5p | TDG | chr12:104382263-104382270[+] | 7[1] | 0[0] | 0[0] | 7[1] | 166[7] | 3 | 3 |
| hsa-miR-30e-5p | PSMD7 | chr16:74340146-74340152[+] | 1933[20] | 0[0] | 0[0] | 1933[20] | 1933[20] | 3 | 3 |
| hsa-miR-30e-5p | ARID5B | chr10:63853572-63853579[+] | 275[9] | 275[9] | 0[0] | 275[9] | 366[9] | 4 | 3 |
| hsa-miR-30e-5p | PAWR | chr12:79986031-79986038[-] | 525[12] | 0[0] | 0[0] | 525[12] | 525[12] | 3 | 5 |
| hsa-miR-30e-5p | CCDC6 | chr10:61552240-61552246[-] | 550[12] | 0[0] | 0[0] | 550[12] | 557[12] | 3 | 3 |
| hsa-miR-30e-5p | NDEL1 | chr17:8371251-8371258[+] | 4013[20] | 0[0] | 0[0] | 4013[20] | 13506[23] | 3 | 10 |
| hsa-miR-30e-5p | HSPA5 | chr9:127998723-127998729[-] | 1003[5] | 0[0] | 0[0] | 1003[5] | 1004[8] | 3 | 5 |
| hsa-miR-30e-5p | RAD23B | chr9:110092635-110092642[+] | 421[9] | 0[0] | 0[0] | 421[7] | 450[11] | 3 | 6 |
| hsa-miR-30e-5p | RAD23B | chr9:110092687-110092693[+] | 556[8] | 0[0] | 0[0] | 556[8] | 556[10] | 3 | 6 |
| hsa-miR-30e-5p | IRF2BP2 | chr1:234740285-234740291[-] | 102[7] | 102[7] | 0[0] | 0[0] | 102[7] | 3 | 3 |
| hsa-miR-30e-5p | CPEB4 | chr5:173385991-173385998[+] | 195[5] | 0[0] | 0[0] | 195[5] | 196[6] | 3 | 3 |
| hsa-miR-30e-5p | RAP2C | chrX:131338458-131338464[-] | 85[7] | 0[0] | 0[0] | 85[7] | 87[8] | 3 | 5 |
| hsa-miR-30e-5p | RAP2C | chrX:131339122-131339128[-] | 9[5] | 0[0] | 0[0] | 9[5] | 11[6] | 3 | 5 |
| hsa-miR-30e-5p | PRPF40A | chr2:153512797-153512804[-] | 119[5] | 0[0] | 0[0] | 119[5] | 119[5] | 3 | 3 |
| hsa-miR-30e-5p | RNF122 | chr8:33405721-33405728[-] | 33[8] | 0[0] | 0[0] | 33[8] | 38[9] | 3 | 4 |
| hsa-miR-30e-5p | RTN4R | chr22:20229064-20229071[-] | 208[8] | 0[0] | 0[0] | 208[8] | 208[8] | 3 | 3 |
| hsa-miR-30e-5p | KLF10 | chr8:103661032-103661039[-] | 2050[19] | 0[0] | 0[0] | 2050[19] | 2121[24] | 3 | 9 |
| hsa-miR-30e-5p | MYO5A | chr15:52605184-52605191[-] | 79[7] | 79[7] | 0[0] | 79[7] | 79[7] | 4 | 8 |
| hsa-miR-30e-5p | ABL1 | chr9:133763049-133763056[+] | 514[7] | 514[7] | 0[0] | 514[7] | 519[8] | 4 | 6 |
| hsa-miR-30e-5p | CBX3 | chr7:26252912-26252918[+] | 278[9] | 0[0] | 0[0] | 278[9] | 582[11] | 3 | 3 |
| hsa-miR-30e-5p | GALNT1 | chr18:33289962-33289969[+] | 1075[12] | 0[0] | 0[0] | 1075[12] | 1075[12] | 3 | 6 |
| hsa-miR-30e-5p | KLHL20 | chr1:173755693-173755700[+] | 278[7] | 0[0] | 0[0] | 278[7] | 278[7] | 3 | 4 |
| hsa-miR-30e-5p | KLHL20 | chr1:173755753-173755760[+] | 169[9] | 0[0] | 0[0] | 169[9] | 225[9] | 3 | 4 |
| hsa-miR-30e-5p | CAPZA1 | chr1:113213939-113213946[+] | 2301[19] | 0[0] | 0[0] | 2301[19] | 2301[19] | 3 | 5 |
| hsa-miR-30e-5p | KIAA1211 | chr4:57196457-57196463[+] | 150[7] | 0[0] | 0[0] | 150[7] | 163[7] | 3 | 3 |
| hsa-miR-30e-5p | WDR44 | chrX:117583713-117583720[+] | 97[4] | 0[0] | 0[0] | 97[4] | 213[5] | 3 | 5 |
| hsa-miR-30e-5p | CPEB2 | chr4:15068742-15068748[+] | 587[5] | 587[5] | 0[0] | 587[5] | 587[7] | 4 | 4 |
| hsa-miR-30e-5p | BECN1 | chr17:40962673-40962680[-] | 68[4] | 0[0] | 0[0] | 68[4] | 350[8] | 3 | 6 |
| hsa-miR-30e-5p | PIP4K2A | chr10:22825027-22825034[-] | 593[8] | 593[8] | 0[0] | 593[8] | 597[8] | 4 | 4 |
| hsa-miR-30e-5p | PIP4K2A | chr10:22826007-22826014[-] | 164[10] | 164[10] | 0[0] | 164[10] | 164[10] | 4 | 4 |
| hsa-miR-30e-5p | MTDH | chr8:98737185-98737192[+] | 2525[7] | 2525[7] | 0[0] | 2525[7] | 0[0] | 3 | 6 |
| hsa-miR-30e-5p | FLJ36031 | chr7:106300544-106300551[-] | 1180[12] | 0[0] | 0[0] | 1180[12] | 1180[12] | 3 | 8 |
| hsa-miR-30e-5p | KPNA6 | chr1:32637378-32637384[+] | 732[16] | 0[0] | 0[0] | 732[16] | 756[17] | 3 | 4 |
| hsa-miR-30e-5p | PICALM | chr11:85668788-85668795[-] | 1608[14] | 0[0] | 0[0] | 1608[14] | 1608[14] | 3 | 7 |
| hsa-miR-30e-5p | DCP1A | chr3:53319274-53319281[-] | 148[7] | 0[0] | 0[0] | 148[7] | 157[8] | 3 | 4 |
| hsa-miR-30e-5p | PTP4A1 | chr6:64290580-64290587[+] | 6747[20] | 0[0] | 0[0] | 6747[20] | 6747[20] | 3 | 7 |
| hsa-miR-30e-5p | PTP4A1 | chr6:64291015-64291021[+] | 1291[2] | 0[0] | 0[0] | 1291[2] | 1715[16] | 3 | 7 |
| hsa-miR-30e-5p | PHTF2 | chr7:77584309-77584316[+] | 396[9] | 0[0] | 0[0] | 396[9] | 404[9] | 3 | 6 |
| hsa-miR-30e-5p | PAPD5 | chr16:50265250-50265256[+] | 89[8] | 0[0] | 0[0] | 89[8] | 99[9] | 3 | 4 |
| hsa-miR-30e-5p | RAB11A | chr15:66180428-66180434[+] | 336[5] | 0[0] | 0[0] | 168[5] | 336[5] | 3 | 3 |
| hsa-miR-30e-5p | ITSN1 | chr21:35210340-35210347[+] | 160[5] | 0[0] | 0[0] | 160[5] | 800[5] | 3 | 7 |
